# Oxidative Stress Disrupted Prepubertal Rat Testicular Development after Xenotransplantation

**DOI:** 10.1155/2021/1699990

**Published:** 2021-11-17

**Authors:** Yu-Bo Ma, Ming Gao, Tong-Dian Zhang, Tie Chong, He-Cheng Li, Zi-Ming Wang, Lian-Dong Zhang

**Affiliations:** ^1^Department of Urology, The Second Affiliated Hospital of Xi'an Jiaotong University, Xi'an, Shaanxi 710004, China; ^2^Department of Nephrology, Xi'an No. 4 Hospital, Xi'an, Shaanxi 710004, China; ^3^Department of Andrology, Liaocheng People's Hospital, Liaocheng, Shandong 252000, China

## Abstract

In the past two decades, testicular tissue grafting and xenografting have been well established, with the production of fertilization-competent sperm in some studies. However, few studies have been carried out to observe the development of grafted prepubertal testicular tissue of rats and compare the biological differences between in situ testis and grafted testis. In this study, we established the prepubertal testicular tissue xenografting model using a 22-day-old rat and evaluated certain parameters, including testicular histology, testosterone production, and ultrastructure of the grafted testes. We also assessed gene expression of cell proliferation markers, testicular cell markers, and antioxidative defense system. Our results showed that 47 days after transplantation, intratesticular testosterone concentration was not significantly altered; however, cell proliferation, spermatogenesis, and Sertoli cell markers in the transplanted testes were significantly disrupted compared with the control group, accompanied by aggravated apoptosis and oxidative damage. Moreover, the transplanted testes showed smaller tubular diameter and disrupted spermatogenic epithelium with apparent vacuoles, distorted and degenerated germ cells with obscure nuclear margin, and no spermatids in the center of the tubules. Although testis xenografting has been extensively tested and attained great achievement in other species, the prepubertal rat testicular tissue xenografting to immunodeficient mice exhibited obvious spermatogenesis arrest and oxidative damage. The protocol still needs further optimization, and there are still some unknown factors in prepubertal rat testes transplantation.

## 1. Introduction

Owing to remarkable progress in the treatment of childhood cancer in recent years, we have seen an increasing number of long-term survivors whose five-year survival rate for all cancers (combined) was 81% in children and 87% in adolescents [1]. Improvement of long-term survival rate is of great significance; however, there is a huge price hidden behind this achievement. Chemotherapy and radiation treatments for cancer can deplete spermatogonial stem cells (SSCs) in the testis, resulting in irreversible infertility [2]. Therefore, impaired fertility is another life crisis that these young individuals must confront, besides cancer itself, and infertility might play an important role in affecting the psychological aspects of their lives [3]. Before undergoing gonadotoxic treatment, adult men can cryopreserve their sperm for future use in assisted reproductive technologies. However, this type of cryopreservation is not an option for prepubertal boys, who are not yet producing sperm at this stage; therefore, preservation of fertility in prepubertal cancer patients has become an important issue [4].

In 1974, Povlsen et al. first transplanted 14- to 22-week-old human fetal organs into nude mice subcutaneously and found fetal testis development after transplantation [5]. However, the study did not attract extensive concern until Honaramooz et al. observed the establishment of complete spermatogenesis after grafting neonatal testis tissue into mouse hosts in 2002 [6]. Moreover, testicular tissue xenografting was used as a model to evaluate gonad toxicity of endocrine-disrupting chemicals and the translation to humans may offer hope for new strategies to treat male infertility [7]. Mitchell et al. reported that exposure of human fetal testis to di-*n*-butyl phthalate induced no obvious effect on testosterone production by xenografting testicular tissue into castrated male nude mice, which differed greatly from the effect of di-*n*-butyl phthalate exposure on rats [8]. Compared with in vitro fertility preservation methods, testicular transplantation showed advantages of preserving SSCs in the intact testicular niche as well as an established endocrine axis between the host mouse and transplanted tissue, with promising advances towards clinical application [9].

In the past two decades, immature tissue grafting and ectopic xenografting under the skin have been well established. In some studies, when immature testicular tissues from mice, pigs, goats, and monkeys were transplanted under the dorsal skin of immunodeficient nude mice, fertilization-competent sperm was produced and live offspring was generated [6, 10–12]. The status of the donor and the recipient have been proven to influence the outcome of transplantation. Some studies have highlighted the effect of donor age and recipient hormone status on graft survival and development. Compared with immature testicular tissue, the adult testicular tissue transplant usually showed poor outcomes due to its sensitivity to ischemia and hypoxia during the grafting procedure [13]. Different prepubertal donor ages were also proven to affect graft outcome, for example, testicular tissue from a 6-month-old lynx survived better than those from perinatal and 2-year-old lynx after xenografting [14]; therefore, it is still necessary to understand whether prepubertal testes of different stages may exhibit different results after transplantation. Generally, castrated immunodeficient mice were chosen as the transplantation host; however, in some cases, it was found that castration of mice before the transplantation did not modify the outcome of pig testis xenografts [15], and spermatogenic arrest was observed in buffalo testis tissue grafts [16].

Although extensive studies have been carried out to explain the outcome of testicular tissue transplantation, only some have observed the development of grafted prepubertal rat testicular tissue and compared the biological difference between in situ and grafted testes. In this study, we established the prepubertal testicular tissue xenografting model using 22-day-old rats and evaluated parameters including testicular histology, testosterone production, and ultrastructure of the grafted testes. The gene expression of testicular cell markers and antioxidative defense system was also evaluated so as to gain insights into the fertility restoration strategies and the immature testis developmental pattern in different species.

## 2. Materials and Methods

### 2.1. Animals and Xenografting

Prior to initiation of the study, the research protocol was reviewed and approved by the Committee on Animal Research and Ethics of Xi'an Jiaotong University (Xi'an, China).

**Figure 1:**
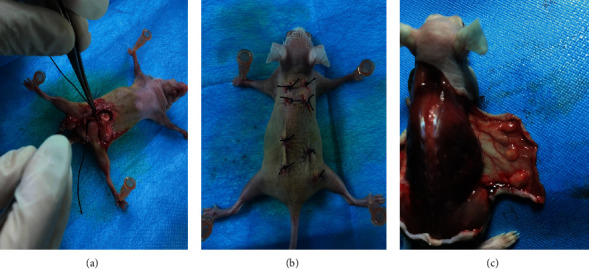
The model of xenotransplantation of testicular tissue: (a) male nude mice were castrated under anesthesia; (b) xenograft was performed 2 weeks after castration; (c) grafted testes were resected on 47th day after xenotransplantation.

Six specific pathogen-free (SPF) BALB/c male nude mice aged 4–5 weeks were purchased from Beijing Vital River Laboratory Animal Technology Co., Ltd., Beijing, China, and were acclimated for 5 days in Experimental Animal Center of Xi'an Jiaotong University. After acclimation, the nude mice were castrated under anesthesia, and xenograft was performed 2 weeks after castration (Figure 1(a)).

Pregnant SPF Sprague–Dawley rats were obtained from the Experimental Animal Center of Xi'an Jiaotong University. On postnatal day 22 (PND 22), male offspring of these rats were anesthetized by intraperitoneal injection of 2% sodium pentobarbital at a dose of 40 mg/kg body weight (Sigma-Aldrich Inc., St. Louis, USA) and then hemicastrated. The left testes were removed aseptically and placed immediately on ice for xenografting. The surgical wound was then sutured, and these male rats were kept as control until PND 69. The left testes that were placed on ice were sliced into small pieces (1–2 mm^3^) and transplanted under the dorsal skin of nude mice (Figure 1(b)). Three or four grafts per rat were transplanted to one side of the back of nude mice, and antibiotics were given in drinking water for 3 days. All experimental animals were treated with purified water and food on an ad libitum basis under a 12 h light/dark cycle.

The grafted testes on the dorsal skin of the nude mice were resected on the 47th day after xenotransplantation (Figure 1(c)). On the same day, the right testes of the male rats in the control group were harvested. Part of the tissues was fixed for histology and ultrastructural study, and the rest was frozen under -80°C for gene analysis and intratesticular testosterone analysis.

### 2.2. Testicular Histology and Staging Spermatogenesis

Following fixation in 4% paraformaldehyde fixative solution at 4°C for 6 h, testicular tissue was transferred to ethanol and xylene, embedded in paraffin, and cut into 5 *μ*m sections. The sections were stained with 0.2% (*w*/*v*) hematoxylin for 3 min and 0.5% (*w*/*v*) eosin for 6 min and evaluated under light microscopy. Spermatogenesis stages were evaluated after hematoxylin and eosin (H&E) staining and classified into early (stages I–VI), mid (stages VII–VIII), and late (stages IX–XIV) stages. The stages were determined considering certain characteristics, such as changing shape and position of the elongated spermatid in the early stages, the size and position of the residual body for mid stages, and the shape and morphology of the elongating spermatid to identify the late stages. These evaluations were performed by an independent investigator blind to treatment.

### 2.3. 8-OH-dG Detection

After antigen retrieval and endogenous peroxidase blocking, the sections were incubated at 4°C overnight with anti-8-OH-dG polyclonal antibody (1 : 500, cat# bs-1278R, Beijing Biosynthesis Biotechnology Co., Ltd., China) in a humidified chamber, followed by conjugation to the goat anti-rabbit secondary antibody (cat# SP-0023, Beijing Biosynthesis Biotechnology Co., Ltd., China) and 3,3′-diaminobenzidine (cat# C-0010, Beijing Biosynthesis Biotechnology Co., Ltd., China) staining. The negative control was established with the primary antibody replaced by phosphate-buffered saline (PBS).

### 2.4. Terminal Deoxynucleotidyl Transferase dUTP Nick-End Labeling Assay

Terminal deoxynucleotidyl transferase (TdT) dUTP nick-end labeling assay (TUNEL) was performed using a TUNEL Apoptosis Assay Kit (cat# C1098, Beyotime Biotechnology Co., Ltd., China) according to the manufacturer's instructions. In brief, the sections were deparaffinized, hydrated, and incubated with 20 *μ*g/mL DNase-free Proteinase K at 37°C for 20 min. After washing with PBS and incubation with 3% H_2_O_2_ in PBS at 25°C for 20 min, the sections were incubated with working solution containing TdT enzyme and Biotin-dUTP at 37°C in the dark for 60 min. Next, after washing with PBS, the sections were incubated with streptavidin-horse radish peroxidase solution, followed by DAB working solution. Negative control was set according to the manufacturer's instructions. Seminiferous tubules containing two or more TUNEL-positive cells were counted as positive. The apoptosis index (AI) was calculated as the ratio of number of positive tubules of apoptosis and total number of tubules in a cross section.

### 2.5. Ultrastructural Study

The harvested tissue were promptly washed with 0.1 mol/L PBS and immersed in 4% (*w*/*v*) formaldehyde and 2.5% (*w*/*v*) glutaraldehyde in 0.1 mol/L PBS for 2 h at 4°C. Then, tissue was postfixed in 1% (*w*/*v*) osmium tetroxide for 2 h in 0.1 mol/L PBS at 4°C for 1 h. After dehydrating, embedding, and sectioning, the sections were double stained with uranyl acetate for 15 min and lead citrate for 5 min. The sections were then observed under an H-7650 transmission electron microscope at 80 kV (Hitachi, Japan).

### 2.6. Intratesticular Testosterone Analysis

Testicular tissue was weighed and then homogenized in 0.2 mL ice-cold normal saline using an Ultra-Turrax (T8; IKA®-Werke GmbH & Co., KG, Staufen, Germany). Subsequently, testicular homogenates were centrifuged at 3000 rpm for 15 min at 4°C, and then, the supernatant was collected. The intratesticular testosterone concentration was measured using the Elecsys Testosterone II kit (cat# 05200067190, Roche, Germany) according to the manufacturer's instructions. The intratesticular testosterone concentration was expressed as nanogram per gram (ng/g).

### 2.7. RNA Extraction and Quantitative Real-Time Polymerase Chain Reaction (PCR)

Total RNA was extracted using the TaKaRa MiniBEST Universal RNA Extraction Kit (cat# 9767, Takara, Japan) and converted to cDNA using PrimeScript™ RT Master Mix (cat# RR036A, Takara, Japan). Quantitative real-time PCR was performed using TB Green Premix Ex Taq II (cat# RR820A, Takara, Japan) on the Bio-Rad CFX Connect Real-Time PCR Detection System (Bio-Rad, USA). Glyceraldehyde-3-phosphate dehydrogenase (Gapdh) was used as an endogenous control for normalization. The thermal cycle consisted of initial 2 min at 95°C, followed by 39 cycles of 95°C for 10 s and 60°C for 30 s. All analyses were performed in triplicate samples, and the relative gene expression was analyzed using the 2^−*ΔCt*^ algorithm. The names of genes and primer sequences are listed in Table 1.

### 2.8. Statistical Analysis

Data were expressed as mean ± standard error of mean and analyzed using unpaired two-tailed *t*-test with statistical analysis functions in GraphPad Prism version 8.0 (GraphPad Inc., USA). Differences were considered statistically significant at the probability level of 5% (*P* < 0.05).

## 3. Results

### 3.1. Gene Expression of Sertoli Cell Markers

**Figure 2:**
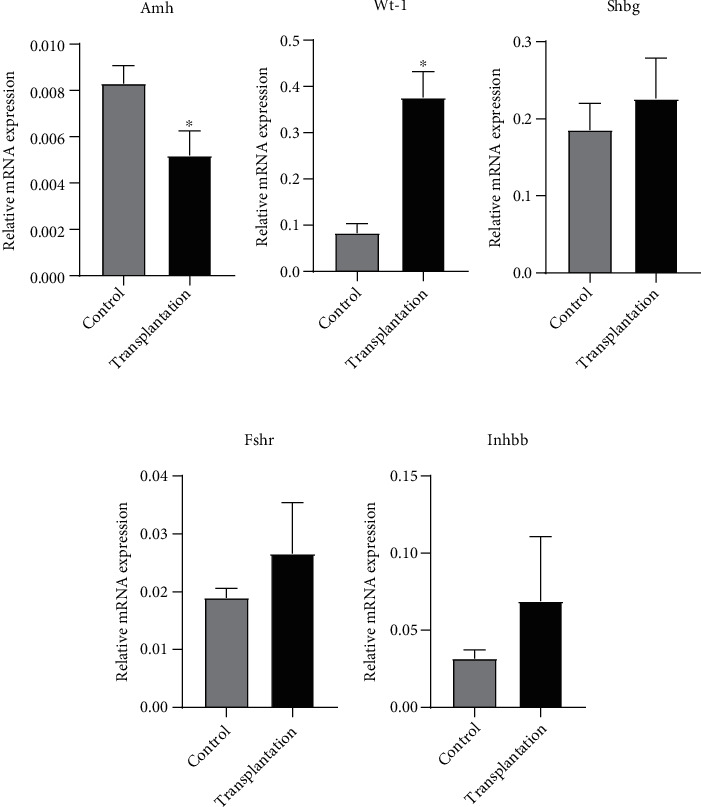
Gene expression of Sertoli cell markers. ^∗^Significantly different from control at *P* < 0.05.

The gene expression of Sertoli cell markers of each group is shown in Figure 2. The expression of Amh in the transplantation group was significantly lower than that in the control group (*P* < 0.05), while the expression of Wt-1 was significantly higher than that in the control group (*P* < 0.05). No significant difference was found in Shbg, Fshr, and Inhbb expression between the two groups (*P* > 0.05).

### 3.2. Gene Expression of Leydig Cell Markers and Concentration of Intratesticular Testosterone

**Figure 3:**
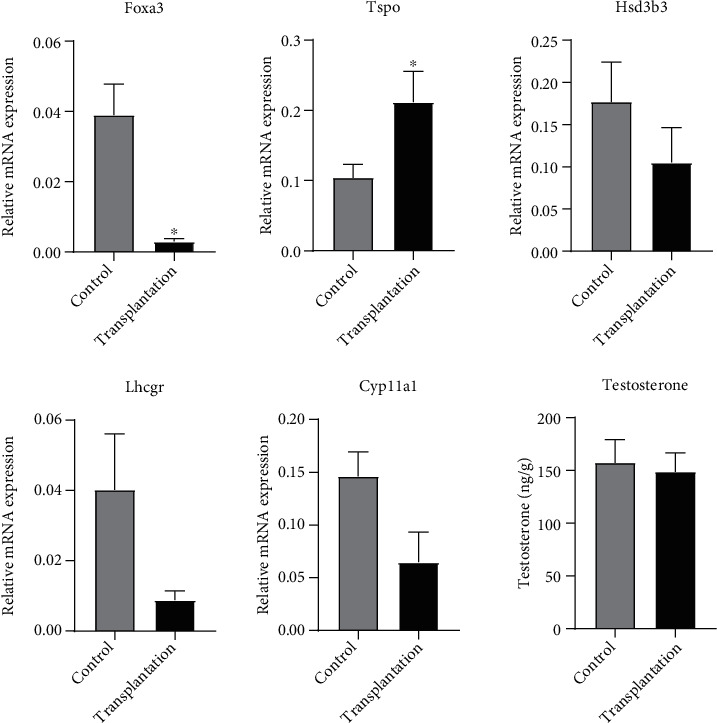
Gene expression of Leydig cell markers and concentration of intratesticular testosterone in control and transplantation groups. ^∗^Significantly different from control at *P* < 0.05.

The gene expression of Leydig cell markers is shown in Figure 3. The expression of Foxa3 in the transplantation group was significantly lower than that in the control group (*P* < 0.05), while Tspo expression was significantly higher than that in the control group (*P* < 0.05). No significant difference was found in Hsd3*β*, Lhcgr, and Cyp11a1 expression between the two groups (*P* > 0.05).

The measured intratesticular testosterone concentration is shown in Figure 3. The intratesticular testosterone concentration of the control group (157.07 ± 31.07 ng/g) showed no statistical difference compared with that of the transplantation group (148.40 ± 36.46 ng/g; *P* > 0.05).

### 3.3. Gene Expression of Mitotic Germ Cell Markers

**Figure 4:**
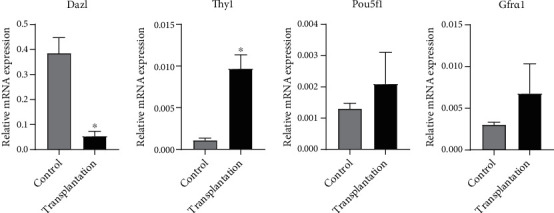
Gene expression of mitotic germ cell markers. ^∗^Significantly different from control at *P* < 0.05.

The expression of mitotic germ cell markers is shown in Figure 4. The expression of Dazl in the transplantation group was significantly lower than that in the control group (*P* < 0.05), while Thy1 expression was significantly higher than that in the control group (*P* < 0.05). No significant difference was found in Gfr*α*1 and Pou5f1 expression between the two groups (*P* > 0.05).

### 3.4. Gene Expression of Meiotic Germ Cell and Spermiogenesis Markers

**Figure 5:**
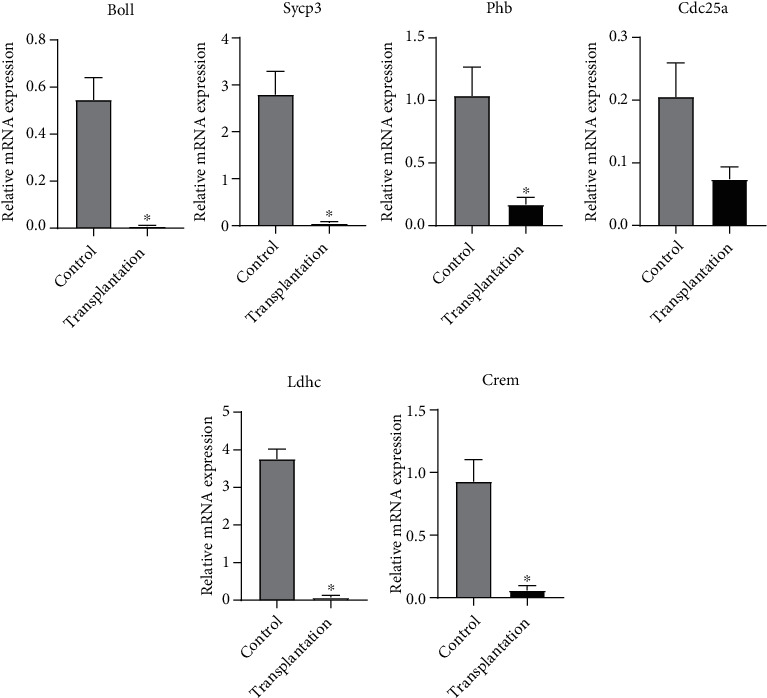
Gene expression of meiotic germ cell (upper row) and spermiogenesis markers (lower row). ^∗^Significantly different from the control at *P* < 0.05.

The gene expression of meiotic germ cell markers is shown in Figure 5. The expression of Boll, Sycp3, and Phb in the transplantation group was significantly lower than that in the control group (*P* < 0.05). There was no significant difference in Cdc25a expression between the two groups (*P* > 0.05). In terms of spermiogenesis markers, the Ldhc and Crem expression levels were significantly lower in the transplantation group than in the control group (*P* < 0.05).

### 3.5. Gene Expression of Methyltransferase

**Figure 6:**
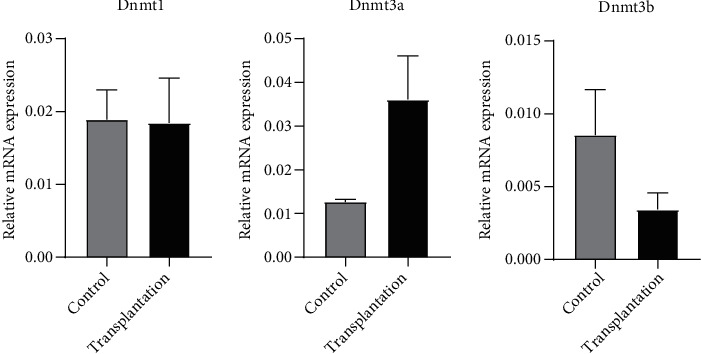
Gene expression of methyltransferase.

The gene expression of methyltransferase is shown in Figure 6. No significant difference was found in Dnmt1, Dnmt3a, and Dnmt3b expression between the two groups (*P* > 0.05).

### 3.6. Gene Expression of Antioxidative Genes

**Figure 7:**
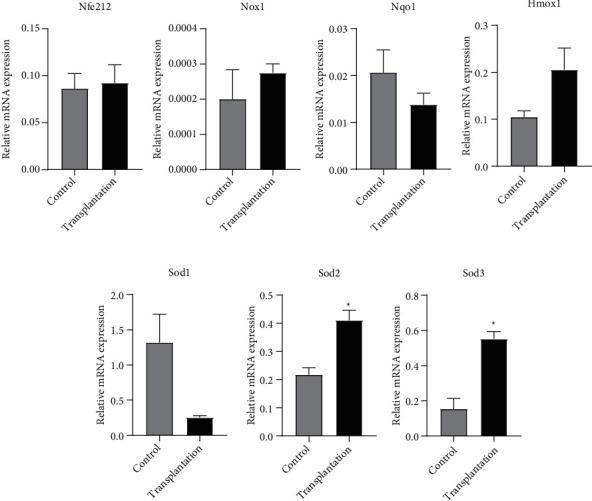
Gene expression of antioxidative genes. ^∗^Significantly different from control at *P* < 0.05.

The expression of antioxidative genes is shown in Figure 7. The expression of Sod2 and Sod3 in the transplantation group was significantly higher than that in the control group (*P* < 0.05). No significant difference was observed in Nfe212, Nox1, Nqo1, Hmox1, and Sod1 expression between the two groups (*P* > 0.05).

### 3.7. Gene Expression of Cell Proliferation Markers

**Figure 8:**
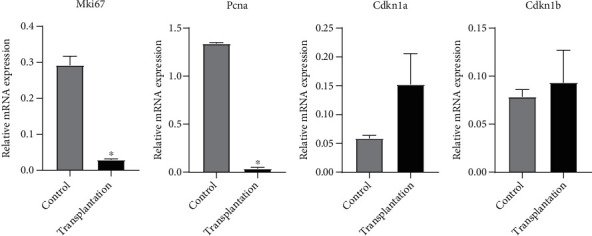
Gene expression of antioxidative genes. ^∗^Significantly different from control at *P* < 0.05.

The gene expression of cell proliferation markers is shown in Figure 8. The expression of Mki67 and Pcna in the transplantation group was significantly lower than that in the control group (*P* < 0.05). No significant difference was observed in Cdkn1a and Cdkn1b expression between the two groups (*P* > 0.05).

### 3.8. Testicular Histology

**Figure 9:**
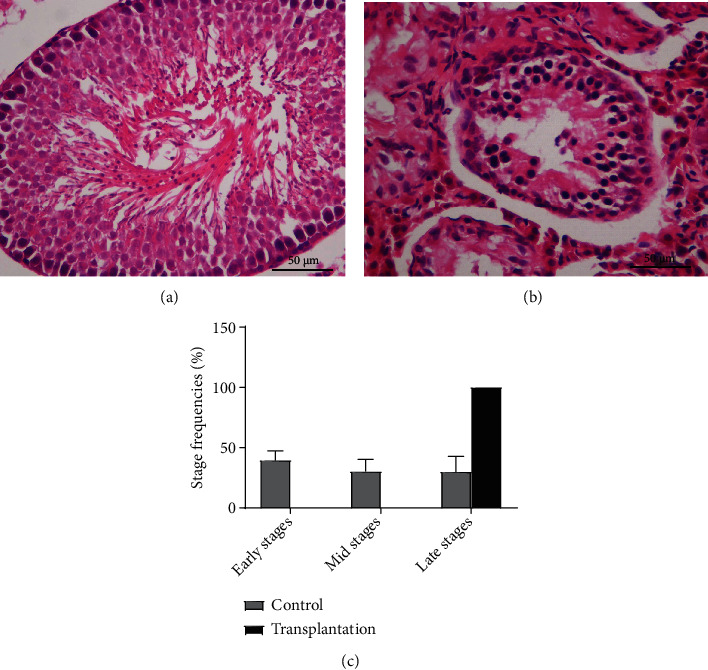
H&E staining of rat testes in control and transplantation groups. H&E staining showed intact testicular structure, and complete spermatogenesis was well established in the control group (a). By contrast, the transplanted testes showed smaller tubular diameter and disrupted spermatogenic epithelium (b). Tubules in the grafted testes were all in the late stages, and spermatogenesis stages in the control group were normally distributed ((c); (a, b): 40x magnification; scale bars indicate 50 *μ*m).

H&E of testicular sections are shown in Figure 9. In the control group, H&E staining showed intact testicular structure without apparent necrosis or vacuoles. Complete spermatogenesis was well established, spermatogonia were seen close to the basement membrane with their dark nuclei, primary spermatocytes were the largest cells, and spermatids appeared smaller than primary spermatocytes and lay near the lumen. By contrast, the transplanted testes showed smaller tubular diameter and disrupted spermatogenic epithelium with apparent vacuoles. The basement membrane was thickened and irregular. Moreover, germ cells in the transplanted testes were loosely arranged, and no spermatids were observed in the center of the tubules. We investigated the stages of spermatogenesis in the transplantation and control groups and found that tubules in the grafted testes were all in the late stages, and spermatogenesis stages in controls were normally distributed, indicating that the prepubertal testis transplantation showed deleterious effects on normal testis development, which may lead to adult spermatogenesis arrest.

### 3.9. Immunohistochemistry of 8-OH-dG

**Figure 10:**
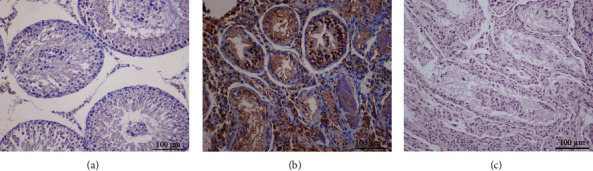
Immunohistochemical staining of 8-OH-dG in (a) control, (b) transplantation, and (c) negative control groups ((a–c): 20x magnification; scale bars indicate 100 *μ*m).

To evaluate the degree of DNA oxidative damage, 8-OH-dG was detected using immunohistochemistry on paraffin sections (Figure 10). In the control group, 8-OH-dG was positive-stained in parts of spermatocytes and interstitial cells. By contrast, testes in the transplantation group were strongly positive-stained in the spermatogenic epithelium, and the tubules were deformed and disorderly arranged, indicating that prepubertal rat testis xenotransplantation for 47 days inevitably disrupted the normal spermatogenesis and development of seminiferous tubules, accompanied with aggravated oxidative DNA damage.

### 3.10. Comparison of TUNEL Assay

**Figure 11:**
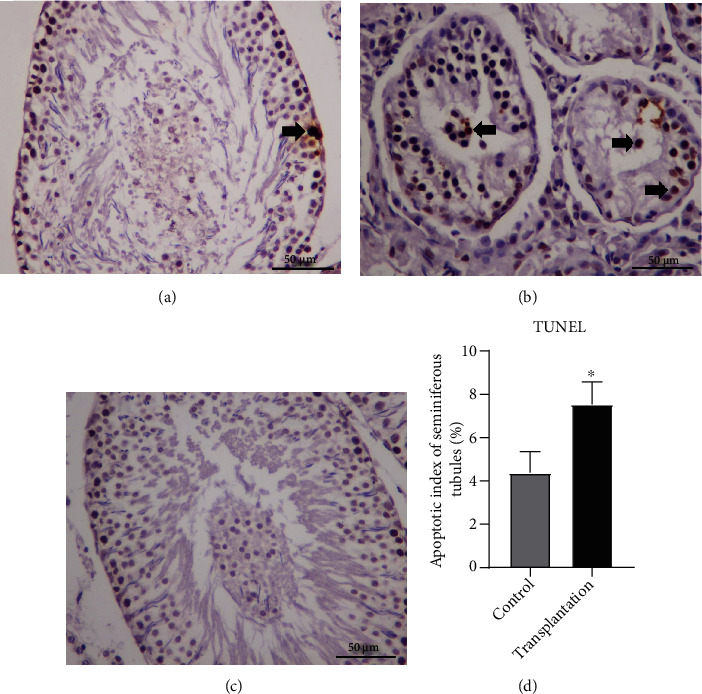
The TUNEL assay in (a) control, (b) transplantation, and (c) negative control groups. The results showed that there were more apoptotic cells (⬆) in the transplantation group than that in the control (d) group ((a, b): 40x magnification; scale bars indicate 50 *μ*m; ^∗^significantly different from control at *P* < 0.05).

The TUNEL assay is shown in Figure 11. The rate of TUNEL-positive cells was generally low in the control group, and the main cell type was spermatogonium. In the transplantation group, more TUNEL-positive germ cells were observed compared with the control group. Moreover, deciduous germ cells were observed in the transplantation group. The irregular seminiferous tubules and AI value in the transplantation group were statistically higher than that in the control group (*P* < 0.05), indicating that oxidative stress may contribute to germ cell apoptosis in the seminiferous epithelium.

### 3.11. Ultrastructural Study

**Figure 12:**
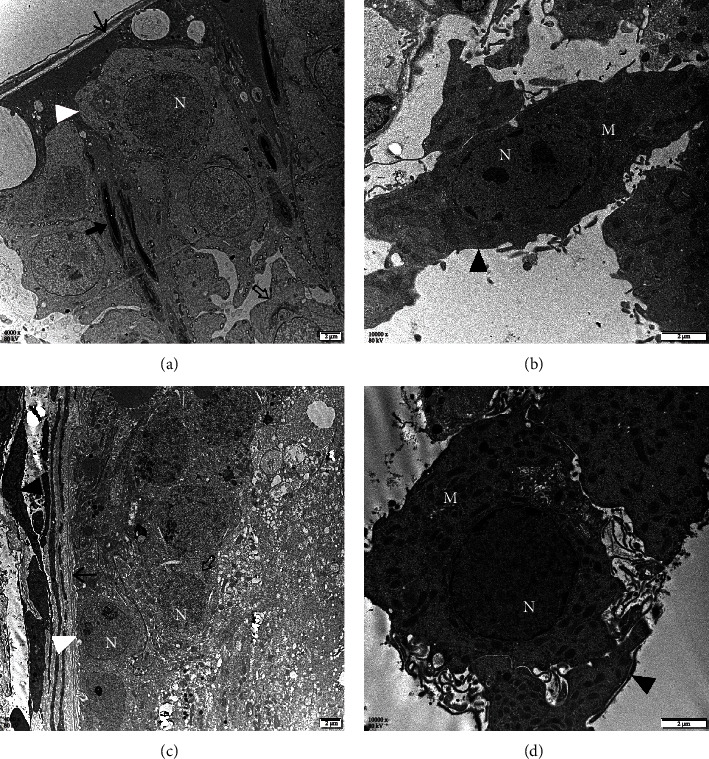
Ultrastructural study of rat testes in (a, b) control and (c, d) transplantation groups (▲: Leydig cell; △: Sertoli cell; ⇧: spermatocyte; ↑: basement membrane; ⬆: elongating spermatids; N: nuclei; M: mitochondria; (a, c): 4000x and (b, d): 10,000x magnification; scale bars indicate 2 *μ*m).

The ultrastructure of testis is shown in Figure 12. In the control group, seminiferous tubules were surrounded by an intact basement membrane, and spermatogenic epithelium was well arranged, which was consistent of spermatogonia, spermatocytes, and spermatids. Sertoli cells were identified by their round, but smaller nucleus with weaker electron density, mostly situated near the basal lamina, and the mitochondria were distributed dispersedly in the cytosol. While in the transplantation group, the basement membrane was loosely arranged and the majority of seminiferous tubules showed degenerative changes. Germ cells were distorted and degenerated with obscure nuclear margin, spermatids were rarely seen, and vacuolation was visible in the spermatogenic epithelium. Both groups had abundant mitochondria in Leydig cells, and no obvious swelling was observed in mitochondria and endoplasmic reticulums. Leydig cells in the transplantation group showed higher electron density in comparison with that of the control group.

## 4. Discussion

With the improvement of cancer therapeutic effects and increase in childhood cancer survival rates, fertility preservation has become an important component of oncologic treatment, especially for those accepting aggressive chemo/radiotherapy [17]. It was found that 46% of all childhood cancer survivors reported infertility, and more than half of the survivors who received alkylating agent chemotherapy had a sperm concentration < 15 million/mL [18, 19]. The disruption of the germ cell population and testicular somatic cells induced by chemotherapeutic drugs/radiation can persist far into adulthood even after treatment cessation. For these reasons, academic societies commonly recommend counseling for pretreatment fertility preservation before the initiation of gonadotoxic therapies [20].

In the past decade, testis tissue grafting has been extensively evaluated in numerous species with variable results; however, many aspects remain unclear due to species difference and complexity of spermatogenesis. In this study, we established the prepubertal rat testicular tissue xenograft model and compared the developmental difference between in situ testis and grafted testis. We found that among Sertoli cell markers, expression of Amh and Wt-1 in the transplantation group was significantly different from that in the control group. Rajpert-De et al. found that the decrease in Amh expression may reflect the terminal differentiation of Sertoli cells and was probably only partially dependent upon a regulatory factor associated with the onset of meiosis [21]. Wt-1 is expressed exclusively by Sertoli cells in the seminiferous epithelium of the adult testis; therefore, Wt-1 knockout resulted in the disruption of developing seminiferous tubules and subsequent progressive loss of Sertoli cells and germ cells. The alternation of Wt-1 in the transplantation group may be responsive upregulation for the maintenance of Sertoli cells and seminiferous tubules in testes [22]. Histologically, disrupted spermatogenic epithelium with apparent vacuoles, along with thickened and irregular basement membrane was observed in the transplantation group. Close and dynamic interactions between germ cells and supporting Sertoli cells are required for the establishment of spermatogenesis. Sertoli cells in the prepubertal period are relatively quiescent, and vacuolation of Sertoli cells is believed to be an early feature of morphological injury, prior to germ cell degeneration [23]. As the tubular vacuoles are usually within or between Sertoli cells, the occurrence of vacuoles is indicative of a breakdown in Sertoli-germ cell junctions and degeneration of germ cells [24].

In this study, we used castrated nude mice as recipients, consistent with previous studies. The removal of the host testes can help monitor androgen production by graft Leydig cells and avoid the interference of host testes with xenografts responding to host gonadotropins. Moreover, removal of the host testes released the negative feedback on the mouse pituitary, and a feedback axis would be reestablished between the grafted tissue and the host hypothalamus and pituitary [25]. In this study, we found that the expression of Foxa3 and Tspo expression was significantly altered in the transplantation group. A previous study revealed that Foxa3 was a testis-specific transcription factor, mainly expressed in Leydig cells [26]. Additionally, Foxa3 knockout subsequently induced several gene alterations in mice, including several interesting testis-specific kallikreins implicated in semen liquefaction and male fertility [26]. Tspo is a high-affinity cholesterol-binding protein, which is abundant in Leydig cells and functions as a cholesterol mitochondrial transporter [27]. The differential expression of Foxa3 and Tspo regulated the testosterone production, and the intratesticular testosterone concentration showed no statistical difference between the two groups, indicating that Leydig cell function was less affected and the hypothalamus-pituitary-testis axis was reestablished after transplantation.

Male germ cells are in intimate contact with somatic cells. Of all the cell types, Sertoli cells are located on the basal lamina of the tubules and surround the germ cells by extending elaborate processes. Peritubular myoid cells, located at the extratubular side of the basal lamina, form tubule walls. Spermatogenesis is a highly orchestrated developmental process that can be divided into three parts: spermatocytogenesis, meiosis, and spermiogenesis. In this study, we found that more than half of the mitosis, meiosis, and spermiogenesis markers were significantly downregulated. Moreover, the gene expression of Mki67 and Pcna in the transplantation group was significantly lower than that in the control group, indicating the suppression of testicular cell proliferation in the transplantation group. Among all cell markers, Dazl is mainly expressed in the early stages of spermatogenesis, with highest levels in pachytene spermatocytes. It was confirmed that disruption of Dazl led to spermatogenesis arrest and loss of germ cells [28]. Boll is a member of the DAZ family, and it plays an important role in testicular function, maintenance, and spermatogenesis. Previous studies have revealed that Boll downregulation was associated with the severity of testicular failure, and loss of Boll may cause male infertility [29]. The other downregulated genes were involved in synaptonemal complex formation (Sycp3), transcriptional regulation (Crem), mitochondrial function regulation (Phb), and sperm motility maintenance (Ldhc), finally leading to testicular degeneration as indicated in the histological findings. Sycp3 is a functional component of the synaptonemal complexes, and it is considered to determine meiotic progression and structural integrity of meiotic chromosomes [30]. Ldhc is testis-specific and plays a vital role for sperm motility by facilitating the conversion of l-lactate and nicotinamide adenine dinucleotide (NAD) to pyruvate and the reduced form of nicotinamide adenine dinucleotide (NADH) [31]. Phb was found negatively correlated with mitochondrial reactive oxidative species (ROS) levels, and loss of Phb in spermatocytes resulted in complete male infertility, associated with apoptosis resulting from mitochondrial morphology and function impairment [32]. Crem is an important component of the cAMP-mediated signaling pathway, which is essential for differentiation of haploid male germ cells, and lack of functional Crem proteins leads to spermiogenesis arrest at the level of round spermatids [33]. In accordance with the gene alternations, we found that prepubertal testis transplantation showed deleterious effects on testis development histologically and ultrastructurally, finally leading to adult spermatogenesis arrest.

Spermatogenesis is a high energy-demanding process, and low levels of oxidative stress are essential for normal testicular function. In the physiological state, testes are equipped with a potent antioxidant system, which protects testes against oxidative injuries [34]. In this study, we found that spermatogenic epithelium staining showed strong positive 8-OH-dG after transplantation. Moreover, more TUNEL-positive germ cells were observed, in addition to deciduous germ cells. The irregular seminiferous tubules indicated that prepubertal rat testis xenotransplantation for 47 days inevitably disrupted the normal spermatogenesis, accompanied with aggravated oxidative damage. Sod2 and Sod3 encode superoxide dismutases, which catalyze the dismutation of superoxide radicals to molecular oxygen and hydrogen peroxide, protecting testicular tissue from oxidative injuries. Although the expression of Sod2 and Sod3 in the transplantation group was significantly higher than that in the control group, the imbalance between the generation and elimination of ROS inevitably disrupted the normal cellular functions and aggravated germ cell apoptosis. Normally, relatively poor vascularization of the testes makes intratesticular oxygen tensions lower than the other parts of the reproductive tracts [35]. However, ischemia and hypoxia are inevitable until a functional circulatory connection is established between host and grafts. It was revealed that a circulatory connection was established between graft and subcutaneous blood vessels by a combination of outgrowing small capillaries from the donor tissue and formation of larger vessels by the host [36]. The lack of uniformity in diffusion and new vessel development could be responsible for the asynchronous development and low efficiency of spermatogenesis.

## 5. Conclusion

In this study, we established the prepubertal rat testis xenografting model and evaluated testicular development after transplantation. Our results revealed that intratesticular testosterone concentration was not significantly altered following transplantation; however, spermatogenesis and Sertoli cell development in the transplanted testes were significantly disrupted, accompanied with aggravated apoptosis and oxidative damage. Although testis xenografting has been extensively tested with great achievement in other species, prepubertal rat testicular tissue xenografting to immunodeficient mice showed obvious oxidative damages and spermatogenesis arrest. The protocol still needs further optimization, and there are still some unknown factors in prepubertal rat testis transplantation, which requires further study.

## Figures and Tables

**Table 1:** The genes and primer sequences.

Gene name	Accession no.	Forward primer	Reverse primer
Gapdh	NM_017008.3	5-GGCACAGTCAAGGCTGAGAATG-3	5-ATGGTGGTGAAGACGCCAGTA-3
Nfe2l2	NM_031789.2	5-ACGTGATGAGGATGGGAAAC-3	5-TATCTGGCTTCTTGCTCTTGG-3
Nox1	NM_053683.1	5-CTCTGCTCCAGAGGAAGAATTT-3	5-CATTGGTGAGTGCTGTTGTTC-3
Nqo1	NM_017000.3	5-GCTGCAGACCTGGTGATATT-3	5-ACATGGTGGCATACGTGTAG-3
Hmox1	NM_012580.2	5-GTCCCTCACAGACAGAGTTTC-3	5-AACTAGTGCTGATCTGGGATTT-3
Sod1	NM_017050.1	5-GGTCCACGAGAAACAAGATGA-3	5-CAATCCCAATCACACCACAAG-3
Sod2	NM_017051.2	5-AGCGTGACTTTGGGTCTTT-3	5-AGCGACCTTGCTCCTTATTG-3
Sod3	NM_012880.1	5-GAGATCTGGATGGAGCTAGGA-3	5-ACCAAGCCTGTGATCTGTG-3
Hsd3b3	NM_001042619.1	5-TTCCTGCTGCGTCCATTT-3	5-GATCTCTCTGAGCTTTCTTGTAGG-3
Lhcgr	NM_012978.1	5-CGCTTCCTCATGTGTAATCTCT-3	5-CCAGTCTATGGCGTGGTTATAG-3
Tspo	NM_012515.2	5-CTATGGTTCCCTTGGGTCTCTA-3	5-AAGCATGAGGTCCACCAAAG-3
Cyp11a1	NM_017286.3	5-AGAACATCCAGGCCAACATC-3	5-CCTTCAAGTTGTGTGCCATTTC-3
Foxa3	NM_017077.2	5-GCTGACCCTGAGTGAAATCTAC-3	5-TCATTGAAGGACAGCGAGTG-3
Amh	NM_012902.1	5-CTAACCCTTCAACCAAGCAAAG-3	5-GGAGTCATCCGCGTGAAA-3
Fshr	NM_199237.1	5-TGTGCCAATCCTTTCCTCTAC-3	5-TGTAAATCTGGGCTTGCATTTC-3
Shbg	|NM_012650.1	5-AAGGACAGAGACTGGACATAGA-3	5-TTAGTGGGAGGTGTGGGTAT-3
Inhbb	NM_080771.1	5-CGAAGGCAACCAGAACCTATT-3	5-TACACCTTGACCCGTACCTT-3
WT-1	NM_031534.2	5-CACCAGGACTCATACAGGTAAA-3	5-TGTTGTGATGGCGGACTAA-3
Dnmt1	|NM_053354.3	5-ACTTTCTCGAGGCCTACAATTC-3	5-TTTCCCTTCCCTTTCCCTTTC-3
Dnmt3a	NM_001003958.1	5-CCACCAGGTCAAACTCCATAAA-3	5-GCCAAACACCCTTTCCATTTC-3
Dnmt3b	NM_001003959.1	5-CGACAACCGTCCATTCTTCT-3	5-GTCGATCATCACTGGGTTACAT-3
Dazl	NM_001109414.1	5-AGTCCAAATGCTGAGACATACA-3	5-TGAACTGGTGAACTCGGATAAG-3
Thy1	NM_012673.2	5-AGAATCCCACAAGCTCCAATAA-3	5-AGCAGCCAGGAAGTGTTT-3
Pou5f1	NM_001009178.2	5-CCCATTTCACCACACTCTACTC-3	5-TCAGTTTGAATGCATGGGAGA-3
Gfra1	NM_012959.1	5-GTGCTCCTATGAAGAACGAGAG-3	5-TGGCTGGCAGTTGGTAAA-3
Boll	NM_001113370.1	5-AACAGCCTGCATATCACTACC-3	5-GCAGATATAGGAATGGAGCAGAA-3
Sycp3	NM_013041.1	5-GAGCCAGAGAATGAAAGCAATC-3	5-GTTCACTTTGTGTGCCAGTAAA-3
Cdc25a	NM_133571.1	5-GTGAACTTGCACATGGAAGAAG-3	5-CTCACAGTGGAACACGACAA-3
Phb	NM_031851.2	5-CATCACACTACGTATCCTCTTCC-3	5-CTTGAGGATCTCTGTGGTGATAG-3
Ldhc	NM_017266.2	5-ATAGGATCCGACTCCGATAAGG-3	5-GCAATGGCCCAAGAGGTATAG-3
Crem	NM_001110860.2	5-GCCAGGTTGTTGTTCAAGATG-3	5-TGTGGCAAAGCAGTAGTAGG-3
Mki67	NM_001271366.1	5-CCGTAGAATTGGCTGGTCTCA-3	5-AGGCTATCAACTTGCTCTGGTT-3
Pcna	NM_022381.3	5-GCCACTCCACTGTCTCCTAC-3	5-CTAGCAACGCCTAAGATCCTTCT-3
Cdkn1a	NM_080782.4	5-CCTAAGCGTACCGTCCAGAG-3	5-GAGAGCAGCAGATCACCAGATTA-3
Cdkn1b	NM_031762.3	5-GATGTAGTGTCCTTTCGGTGAGA-3	5-ACTCCCTGTGGCGATTATTCAA-3

## Data Availability

Data are available upon request.
